# Potential Health and Performance Effects of High-Level and Low-Level Blast: A Scoping Review of Two Decades of Research

**DOI:** 10.3389/fneur.2021.628782

**Published:** 2021-03-10

**Authors:** Jennifer N. Belding, Robyn M. Englert, Shannon Fitzmaurice, Jourdan R. Jackson, Hannah G. Koenig, Michael A. Hunter, Cynthia J. Thomsen, Uade Olaghere da Silva

**Affiliations:** ^1^Defense Health Group, Leidos, San Diego, CA, United States; ^2^Health and Behavioral Sciences Department, Naval Health Research Center, San Diego, CA, United States

**Keywords:** blast, TBI, low-level blast, explosives, overpressure, military, injury, injury - head trauma

## Abstract

Although blast exposure has been recognized as a significant source of morbidity and mortality in military populations, our understanding of the effects of blast exposure, particularly low-level blast (LLB) exposure, on health outcomes remains limited. This scoping review provides a comprehensive, accessible review of the peer-reviewed literature that has been published on blast exposure over the past two decades, with specific emphasis on LLB. We conducted a comprehensive scoping review of the scientific literature published between January 2000 and 2019 pertaining to the effects of blast injury and/or exposure on human and animal health. A three-level review process with specific inclusion and exclusion criteria was used. A full-text review of all articles pertaining to LLB exposure was conducted and relevant study characteristics were extracted. The research team identified 3,215 blast-relevant articles, approximately half of which (55.4%) studied live humans, 16% studied animals, and the remainder were non-subjects research (e.g., literature reviews). Nearly all (99.49%) of the included studies were conducted by experts in medicine or epidemiology; approximately half of these articles were categorized into more than one medical specialty. Among the 51 articles identified as pertaining to LLB specifically, 45.1% were conducted on animals and 39.2% focused on human subjects. Animal studies of LLB predominately used shock tubes to induce various blast exposures in rats, assessed a variety of outcomes, and clearly demonstrated that LLB exposure is associated with brain injury. In contrast, the majority of LLB studies on humans were conducted among military and law enforcement personnel in training environments and had remarkable variability in the exposures and outcomes assessed. While findings suggest that there is the potential for LLB to harm human populations, findings are mixed and more research is needed. Although it is clear that more research is needed on this rapidly growing topic, this review highlights the detrimental effects of LLB on the health of both animals and humans. Future research would benefit from multidisciplinary collaboration, larger sample sizes, and standardization of terminology, exposures, and outcomes.

## Introduction

Modern explosives have grown increasingly destructive over time and have been identified as the leading cause of injuries in recent conflicts such as Operation Enduring Freedom and Operation Iraqi Freedom (1). In response, advances in personal protective equipment (PPE) and combat casualty care have been prioritized and have resulted in increased rates of survival following blast exposure (2). However, while service members are more likely to survive exposure to blast now than in the past, blast exposure is still associated with significant morbidity and mortality and can result in a variety of adverse health outcomes, including traumatic amputations, traumatic brain injury (TBI), posttraumatic stress disorder, and chronic pain (3, 4). Because estimates suggest that 10–20% of veterans returning from deployment have sustained a TBI, the majority of which are the direct result of blast exposure (5), TBI in particular has received a great deal of attention as a threat to service member health and well-being (6).

When most people think of explosive blasts, the image that comes to mind is a high-level blast (HLB), such as the detonation of an improvised explosive device in combat or a terrorist attack such as the Boston Marathon Bombing in 2013. In such cases, explosives emit a blast wave which causes a transient increase in overpressure, followed by a negative pressure phase, before returning to ambient pressure. During this process, those that are nearby can be subject to injury through five mechanisms: the overpressure wave (i.e., primary blast injury), debris (i.e., secondary blast injury), physical displacement of one's body (i.e., tertiary blast injury), heat and toxins (i.e., quaternary blast injury), and environmental contaminants (i.e., quinary blast injury) (7–9).

One type of blast overpressure that is still insufficiently understood by the scientific community is a commonly underestimated form of blast exposure: low-level blast (LLB). Although there is no clear consensus regarding the distinction between HLB and LLB, subject matter experts (SMEs) agree that an LLB is a form of overpressure that is typically below the strength or intensity of an HLB and can result from firing heavy caliber weapons (e.g., Carl Gustaf bazooka, a Howitzer cannon). Empirical research into this form of overpressure dates back to the early 1980's, when researchers examined the association between LLB and pulmonary injury (10). Research on LLB was reinvigorated in the 2000's, when military operators, particularly instructors of advanced training courses (e.g., breacher training courses), reported that they were experiencing a number of symptoms typically associated with concussion and expressed concerns about their long-term health and well-being following such exposures (11).

With increased recognition of the potential adverse health outcomes associated with both HLB and LLB, our understanding of the effects of blast overpressure has advanced remarkably over the past 20 years. Due to the complexity of explosions themselves, as well as the diverse nature of their potential ramifications for health, a full understanding of blast and its effects requires the utilization of multiple disciplinary perspectives and approaches (8, 9, 12–15). For example, physicists study shock waves and how they differ across environments (16). Engineers develop PPE and gauges to measure blast overpressure (17). Medical providers and researchers investigate the effects of blast on various systems within the human body (e.g., neurological, pulmonary, or auditory systems) (8). Much of this health-focused research in particular is conducted with the ultimate goal of identifying avenues for screening, mitigation, and treatment of blast-induced injuries.

Unfortunately, findings from blast research often fail to cross discipline boundaries and instead remain siloed; most literature reviews on blast exposure tend to focus on a single topic or injury (e.g., blast-induced TBI, blast-induced ocular injury) (12, 18–20). Due to the nature of blast exposure and its potential consequences, multidisciplinary efforts will be required to prevent, mitigate, and treat those exposed to blast. However, developing a truly comprehensive understanding of blast exposure is a near-Herculean effort that requires extensive subject matter expertise across a wide variety of disciplines. Thus, there is a need for a recent, accessible, and comprehensive review of the scientific literature exploring the effects of blast overpressure exposure on health and performance.

The purpose of this review was to identify research on the health-related consequences of overpressure exposure that has been published in peer-reviewed outlets and is publicly accessible to the scientific community. Specifically, we present findings from a scoping review with two complementary foci. The first goal was to provide a broad overview of the interdisciplinary nature of blast research generally; the second was to thoroughly summarize research examining LLB that has been published within the past two decades. We believe this dual-focus approach was necessary due to a lack of consistency in the definition of LLB and related terms, which muddles the distinction between HLB and LLB; this might have resulted in failure to identify relevant literature through database searches if only LLB-related terms had been used. Although there is a critical gap in the literature regarding the distinction between HLB and LLB that desperately needs to be addressed, any such discussion requires a paper of its own and is beyond the scope of this paper.

Due to the particularly broad and complex nature of blast research, which uses a variety of sophisticated designs with diverse populations, we opted to conduct a scoping review rather than a traditional systematic literature review. Briefly, a scoping review is a type of systematic literature review that is designed to map an existing body of diverse literature to characterize the extent, range, and nature of research activities within a topic area (21). Whereas, traditional systematic literature reviews were designed to provide thorough summaries of results bearing on a specific research question addressed with a specific methodology, scoping reviews are used to provide an overview of research on broad topics that may have been addressed with a variety of methodologies. Due to the breadth and diversity of the literature surveyed, scoping reviews are not designed to assess the risk of bias within individual studies or to aggregate principal summary measures or estimates of effect sizes (e.g., odds ratios). However, scoping reviews are more appropriate than traditional systematic literature reviews when the purpose of the review is to characterize large bodies of relevant literature across diverse sources, topic areas, and research designs (22).

## Materials and Methods

Established guidelines (PRISMA-ScR) were followed to ensure methodological rigor (23).

### Data Sources

In January 2019, we conducted a literature search to identify all existing peer-reviewed journal articles pertaining to blast injury and/or exposure that may have implications for health and well-being. We searched PubMed for articles published between January 2000 and January 2019 using the following comprehensive list of search terms: blast injury(ies), blast exposure(s), blast wave(s), as well as the co-occurrence of blast with each of the following terms: bullets, wounds, low-level, low pressure, low intensity, lung, force, trauma, traumatic, concussion, induced, pressure, overpressure, and over pressure (e.g., “blast and bullets,” “blast and wounds”). All duplicates and non-English language articles were removed prior to evaluation for relevance and data extraction.

### Study Selection and Data Extraction

Each identified article from the PubMed search was subject to a progressive three-level review process. Each level was completed by two members of the research team. During the first level of review, one of two independent reviewers examined the title and abstract to determine if the article discussed a topic related to overpressure. If an article was deemed relevant, a level 2 review was performed in which the reviewers extracted information from the abstract regarding the type of overpressure (HLB vs. LLB), study population, scientific discipline of researchers (for human studies only), and the medical specialty discussed (if relevant), using a standardized data extraction form in DistillerSR (Evidence Partners, Ottawa, Canada; see Table 1). Articles deemed relevant to LLB received a full-text level 3 review in which two reviewers independently reviewed and extracted data using a standardized form in Microsoft Word (see Supplementary Material).

**Table 1:** Form used for data extraction at level 1 and level 2.

**Question**	**Response options**	**Multiple responses allowed?**
**Level 1**		
Was this paper relevant to overpressure?	Yes No	No
**Level 2**		
What type of overpressure was discussed?	Acute high-level blast Repetitive low-level blast	Yes
What was the study population?	Live humans Animals Literature review None of the above	Yes
If the research involved humans, what was the researchers' scientific discipline?	Medicine/epidemiology Physics/engineering Other (specify)	Yes
If the discipline was medicine and/or epidemiology, what was the relevant medical specialty discussed?	Neurology (including TBI) Injury (excluding TBI) Pulmonology Mental health Other	Yes

### Interrater Reliability

A random 10% of articles included in levels 1 and 2 were reviewed by both reviewers and inter-rater reliability (IRR) was calculated via Cohen's kappa (24). There was substantial agreement (κ = 0.72) on whether articles were deemed relevant to blast overpressure at level 1 and greater than substantial agreement for the data extracted at level 2 (κ = 0.82) (25). Discrepancies between reviewers were resolved through negotiated consensus. IRR was not calculated for data extracted by the two reviewers at level 3 because the data were primarily qualitative in nature and a direct comparison of reviewers' responses indicated that disagreements were rare; disagreements were resolved through discussion between reviewers.

### Ensuring Comprehensiveness of the Search

After the initial PubMed search was completed, three additional strategies were employed to ensure the comprehensiveness of our search. First, we conducted additional searches in PsycInfo and WebofScience using the search terms previously described. Second, references cited in all papers that received level 3 review were examined for possible relevance. Third, we used Google Scholar to identify papers that referenced the level 3 articles and reviewed their title and abstracts for potential relevance. Data were extracted from all articles identified through this process using the level 3 review process previously described.

## Results

### Level 1 Results

#### Literature Search

**Figure 1:**
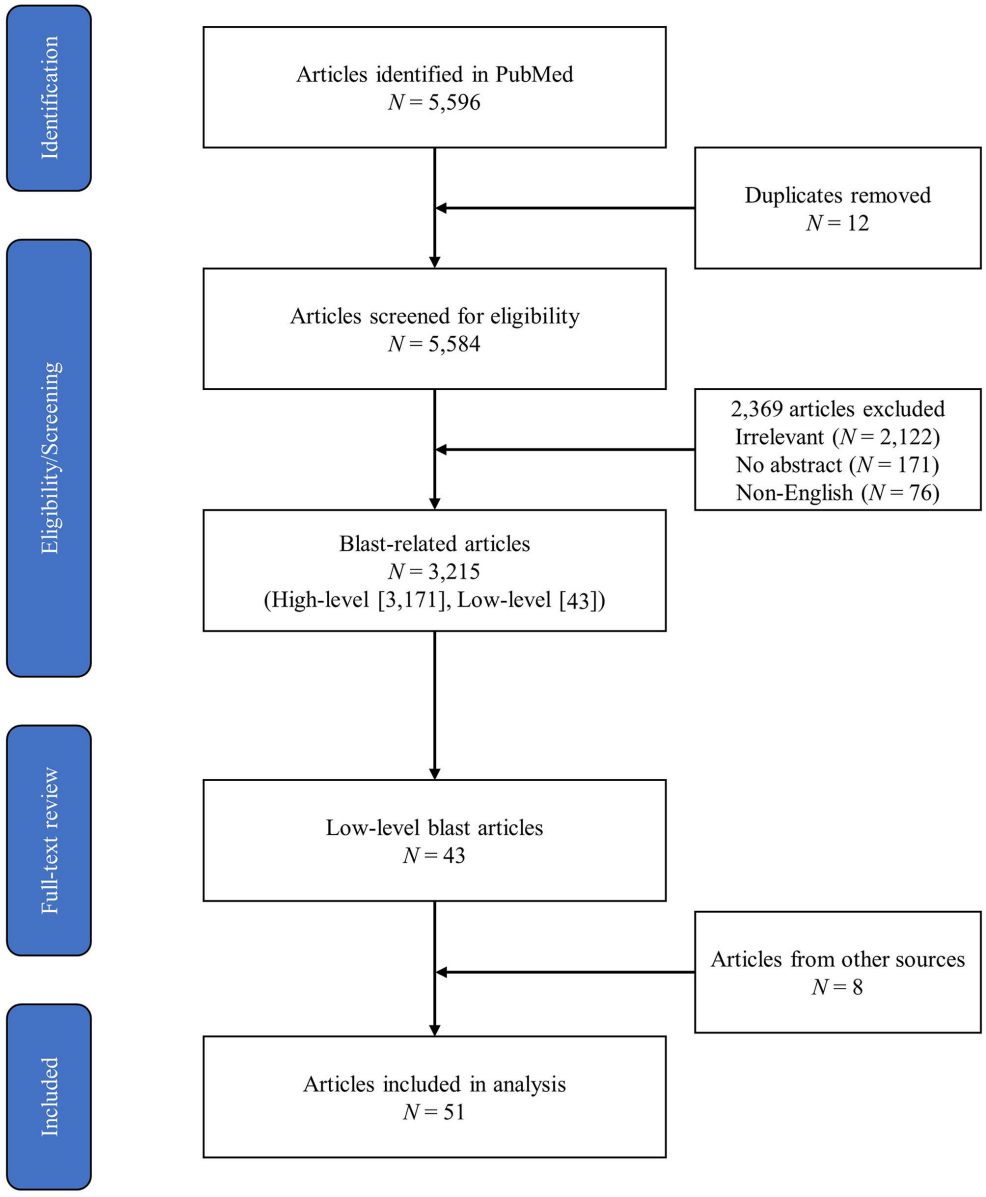
PRISMA-ScR flow diagram.

The initial PubMed search yielded a total of 5,596 peer-reviewed articles, 3,215 of which were identified as relevant to blast overpressure. Forty-three articles from the PubMed search were identified as relevant to LLB, and eight additional articles relevant to LLB were identified through the added literature search methods described previously. This yielded 51 articles that received full-text review and data extraction during level 3. Figure 1 depicts the PRISMA diagram of the literature search process.

### Level 2 Results

#### Blast Overpressure

A summary of the study characteristics extracted from articles during level 2 review are presented in Table 2. Of the 3,215 articles identified as being relevant to blast overpressure, the majority of articles studied live humans (55.37%), while the remainder of the articles were relatively evenly split between those studying animals (16.05%), literature reviews (16.33%), and none of the above (20.16%; e.g., articles on the development of equipment such as helmets that did not involve animals or humans). It is important to note that 6.97% of articles fit into more than one of these categories. Additionally, nearly all research published on blast and identified by our search criteria were conducted by experts working in medicine or epidemiology (99.49%). Only 0.90% of articles were conducted by physicists or engineers and 0.67% were conducted by experts in other disciplines (e.g., education, management, anthropology). Very few articles (*n* = 19; 1.07%) were coded into more than one disciplinary category. Of the 1,771 articles that studied humans and were conducted by researchers within medical or epidemiological fields, approximately half (49.41%) were coded into more than one medical specialty (*M* = 1.56, SD = 0.62). Most published papers focused on non-neurological injury (i.e., injuries excluding TBI; 66.06%), followed by neurology (including TBI; 24.56%), mental health (13.33%), and pulmonology (5.99%). Surprisingly, 45.57% of papers were coded into an “other” category, the majority of which were subsequently identified as articles on management (e.g., hospital staffing required during blast-related mass casualties).

**Table 2:** Frequencies of article characteristics extracted in level 2 review.

	***N***	**%**
**Type of overpressure discussed (*****N*** **=** **3,215)**		
Acute high-level blast	3,198	99.47
Repetitive low-level blast	43	1.34
**Study population (*****N*** **=** **3,215)**		
Live humans	1,780	55.37
Animals	516	16.05
Literature reviews	525	16.33
None of the above	648	20.16
**Scientific discipline (*****N*** **=** **1,780)**		
Medicine/epidemiology	1,771	99.49
Physics/engineering	16	0.90
Other	12	0.67
**Medical specialty (*****N*** **=** **1,771)**		
Neurology (including TBI)	435	24.56
Injury (excluding TBI)	1,170	66.06
Pulmonology	106	5.99
Mental health	236	13.33
Other	807	45.57

*Because response categories were not mutually exclusive, percentages can sum to more than 100%*.

#### LLB

Of the 51 articles identified that addressed LLB, 20 presented empirical data on humans (11, 26–44), 23 presented empirical data on animals (45–66), 1 presented findings from a study developing computational models of LLB exposure (17), and 7 articles were non-empirical (primarily literature reviews or commentaries) (67–73). Table 3 briefly summarizes the essential characteristics of each of the 43 empirical studies, which are further elaborated upon in the Discussion.

**Table 3:** Population, sample size, and setting for empirical studies of low-level blast in humans and animals.

**Citation**	**Population**	***N***	**Setting**
**Human studies**			
Baker et al. (26)	Canadian law enforcement	14	Police explosives technicians – forced entry for instructors course
Blennow et al. (27)	Swedish law enforcement	79	Training exercises
Bonnette et al. (28)	American law enforcement	23	SWAT team training
Capo-Aponte et al. (29)	American military	17	Breacher training course
Carr et al. (31)	American military	40	Breacher training course
Carr et al. (30)	American military and civilian law enforcement	184	Online survey
Carr et al. (32)	American military	108	Breacher training courses
Gill et al. (33)	American military	62	Breacher training courses
Gill et al. (34)	American military	62	Breacher training courses
Kamimori et al. (36)	American military and law enforcement	Not listed	Advanced military and law enforcement training programs
Kamimori et al. (35)	New Zealand military	22	Breacher training course
Kubli et al. (37)	American military	15	Breacher training course
Kulik et al. (38)	Polish civilians	100	Explosives manufacturing company
Littlefield et al. (39)	American military	15	Breacher training course
McBride and Williams (40)	British civilians	357	Electricity transmission company
McBride and Williams (41)	British civilians	357	Electricity transmission company
Rhea et al. (42)	American military	59	Desert warfare training course
St. Onge et al. (43)	American military	Not listed	Breacher training course
Tate et al. (11)	New Zealand military	19	Breacher training courses
Yuan et al. (44)	American law enforcement	18	SWAT team training
**Animal studies**			
Ahlers et al. (45)	Rat	122	Shock tube
Ahmed et al. (46)	Mice	25	Shock tube
Chen et al. (47)	Chinchillas	14	Compressed nitrogen-driven blast apparatus
Choi et al. (48)	Rats	15	Shock tube
Elsayed and Gorbunov (49)	Rats	Not listed	Shock tube
Hall et al. (50)	Rats	70	Shock tube
Li et al. (51)	Rats	10	Shock tube
Lien and Dickman (52)	Mice	Not listed	Shock tube
Park et al. (53)	Rats	124	Shock tube
Perez-Garcia et al. (54)	Rats	Not listed	Shock tube
Perez-Garcia et al. (55)	Rats	28	Shock tube
Por et al. (56)	Rats	25	Shock tube
Pun et al. (57)	Rats	58	Open field explosives
Saljo et al. (59)	Rats	144	Blast tube
Saljo et al. (60)	Rats	180	Shock tube
Saljo et al. (58)	Pigs	115	Various weapons and shock tube
Song et al. (64)	Mice	72	Open field explosives
Song et al. (63)	Mice	20	Open field explosives
Sosa et al. (62)	Rats	Not listed	Shock tube
Sosa et al. (61)	Rats	Not listed	Shock tube
VandeVord et al. (65)	Rats	80	Shock tube
Zuckerman et al. (66)	Rats	98	Exploding wire

### Level 3 Results

#### Overview of HLB

In the past two decades, more than 3,000 articles on the potential health and well-being sequelae associated with blast overpressure have been published, approximately half of which reported data obtained from live human participants. While we anticipated that most of this research would have been published by scholars working in medicine or epidemiology at the outset of this review, we discovered remarkable variability in the medical specialties represented in the literature. Although TBI has been labeled the signature injury of recent conflicts and most deployment-related TBIs are caused by blast (74–76), articles on non-neurological (e.g., traumatic amputation), as opposed to neurological injuries, were published more than twice as often. Articles on mental health emerged frequently, likely because of the comorbidity of TBI and mental health conditions (77–79). It was, however, surprising that nearly half of the medical or epidemiological articles coded were categorized into an “other” category, with hundreds of articles focusing on medical management. These articles tended to focus on the management of healthcare staff and/or facilities (e.g., required trainings, required levels of staffing) as they pertain to treatment of blast-induced injuries (e.g., lessons learned following the Boston Marathon Bombing). Because nearly half of medical/epidemiological studies on humans were coded into more than one subspecialty, our findings demonstrated that health researchers and medical providers from different specialties have begun to collaborate in examining the effects of blast exposure. This contrasts starkly with the general lack of collaboration and cross-talk between members of other scientific disciplines studying the effects of blast exposure. Taken together, evidence from the past two decades clearly shows that HLB exposure can result in injury and/or death, and thus presents a significant threat to warfighter health and well-being.

#### Overview of LLB

Although more than 3,000 articles on blast overpressure have been published since 2000, fewer than 2% of these articles specifically examined health outcomes that may be associated with LLB. Most articles focused on providing empirical data based on animal and human studies, though a few literature reviews and commentaries have been published as well. Our in-depth review of LLB articles revealed substantial diversity in purpose, study design, and conclusions, which we describe subsequently.

#### LLB: Animal Studies

##### Purpose

In general, animal studies of LLB articulated methods to induce blast exposure in well-controlled environments (45–66), examined longitudinal outcomes associated with LLB (46, 64) or repeated exposures to LLB (49), or assessed the impact of environmental characteristics on outcomes associated with LLB (58). Studies frequently examined neurological outcomes, such as neuroinflammation and neurodegeneration (51, 57, 61–63) and damage to specific brain regions or types of cells (53, 65). Animal studies also examined outcomes related to mental health (54, 55) and auditory (47), visual (48, 56), vestibular (52), and vascular (50) systems. Only one study specifically examined an intervention to attenuate adverse outcomes associated with LLB exposure (60).

##### Subjects and Setting

Animal studies used predominately rats (45, 48–51, 53–57, 59–62, 65, 66). Sample sizes in studies using rats ranged from 10 to 180, with 75% of studies including fewer than 100 subjects. Other species studied included mice (46, 52, 63, 64), pigs (58), and chinchillas (47). Sample size for these species varied from 14 (chinchillas) to 115 (pigs). Five studies failed to report a total sample size (49, 52, 54, 61, 62). As expected, all studies using animal subjects were conducted in laboratories.

##### Exposures

In general, LLB exposures were most frequently induced via shock tubes (45, 48, 50, 51, 54, 55, 61) with anesthetized animals (48, 49, 51–57, 61, 62). Most of these studies used the Walter Reed Army Institute of Research shock tube (45, 48, 50, 51, 54, 55, 61) though four studies were conducted at other locations (47, 52, 56, 65). Non–shock tube-induced exposures included subjecting animals to actual weapons systems (e.g., Howitzers, bazookas) (58), explosives (e.g., TNT, C4) (57, 63, 64), and impulse noise (59). In a particularly compelling study of LLB exposure meant to mimic real-world LLB, Zuckerman et al. (66) used an exploding wire technique that allowed for some rats to be subjected to both the psychological stressors and overpressure simultaneously, while others were exposed to only the psychological stressors associated with LLB without the corresponding overpressure. In several studies, animals were exposed to blast only once (45, 52, 65). However, many studies involved repeated exposures (47–49, 54–56, 60, 61) or a combination of single and repeated exposures (56), with the 12 as the highest number of exposures (45, 50). The interval between repeated exposures was most commonly 1 day (45, 48, 50, 54–56, 61), although some induced repeated exposures only minutes (46, 49) or hours apart (47).

In most animal studies of LLB, peak overpressure levels were reported in kilopascals (kPa). There was not only tremendous variability in peak pressures induced, but more importantly, almost all of these exposures exceeded the 4 psi (or ~27.58 kPa) threshold that is often used to differentiate LLB from HLB (35, 36, 46, 67). The peak pressures induced in the lab ranged from 10 kPa (~1.45 psi) (58, 60) to 153 kPa (~22.19 psi) (65), and only three studies meeting our inclusion criteria reported inducing peak pressures below the 4 psi threshold (47, 58, 60).

Studies varied in the physical orientation of the animal relative to the blast, as well as whether restraints were used (e.g., to restrict potential damage from tertiary blast exposure) (46, 49, 50, 54, 57, 61, 65). Animals were typically positioned frontally so the blast reached the head first (51, 54, 55, 60, 61) and were often 1 foot or less from the source of the overpressure (51, 53, 60). Some studies varied, depending on whether animals were restrained as part of the experimental design (45, 56).

##### Outcomes

A wide variety of outcomes were assessed, including measures of physical injury, cognitive and behavioral outcomes, and biomarkers. In terms of general physical outcomes assessed, two studies examined vital signs, such as respiration, heart rate, and body weight, as well as several indicators of neurological injury (e.g., increased intracranial pressure) (46, 58). Additionally, several studies assessed motor dysfunction using rotarod tests (52, 53), balance beam tasks (45), horizontal ladder studies (65), and locomotor activity (55). Further, three studies directly examined injuries in the ocular (56) or auditory systems (47, 52). Behavioral assessments included measures of open-field behavior (53–55, 64), light/dark box behaviors (53, 55, 64), acoustic startle response (55, 66), fear conditioning (55), and a forced swimming test (66). Cognitive outcomes primarily involved assessments of spatial learning and memory, with the Morris water maze test being the most frequently used measure (45, 50, 55, 60, 64–66). There was a remarkable amount of variability in biomarkers assessed (45, 46, 48–53, 56–59, 61–65). including immunohistochemistry assessments for measures of glial fibrillary acidic protein (GFAP), amyloid precursor protein (APP), and more (45, 48, 52, 56, 58, 59, 64). Histopathology was also reported in several studies (45, 51, 57).

##### Summary of Findings From Animal Studies of LLB

Findings from animal studies of LLB have clearly demonstrated that LLB exposure is associated with unique and complex pathological processes (46, 50, 61, 63). Specifically, several studies have demonstrated brain injury as a result of LLB (45, 53, 56, 57, 59, 65), including that associated with neuroinflammation (51, 62), neurodegeneration (59), and white matter perturbations (57). Furthermore, LLB exposure was associated with injury to the visual (48), auditory (47), and vestibular systems (52). Beyond physical injury, LLB exposure was also associated with cognitive and behavioral changes in animals (64), including those resembling PTSD (54, 55, 66, 70) and deficits in learning and memory (45, 50, 65). The effects of repeated LLB exposure in particular are still somewhat mixed in that some studies found greater risk of injury with repeated (vs. single) exposure [e.g., increased learning deficits (45), enhanced pain responses (56), increased activated caspase 3 in the optic nerve (48)], while others have not [e.g., no significant differences in pulmonary-related outcomes (49)]. This suggests that effects of repeated LLB may vary across systems, although more research is needed to conclusively establish these differences. Even so, animal researchers have articulated a need for protection from and prompt treatment for LLB-induced injuries (49).

#### LLB: Human Studies

##### Purpose

The majority of studies examining the effects of LLB on humans attempted to determine if exposure is associated with acute and long-term effects, such as impaired neurological functioning (11, 31, 32, 35, 44); neurochemical evidence of brain damage (27, 32–35); damage to auditory (37, 43), vestibular (26, 39, 43), or visual systems (29); and self-reported symptoms (11, 26, 30, 35). Additionally, one study attempted to quantify and validate the extent of LLB exposure during military and law enforcement training exercises (36). These studies predominantly examined military service members or law enforcement personnel and were primarily observational in nature. Other studies examined outcomes associated with occupational exposure to impulse noise and/or overpressure from explosives (38), exposure to air blast circuit breakers (40, 41), and the effectiveness of specific equipment [e.g., to measure neuromotor function after blast exposure (42), PPE (28, 44)].

##### Subjects and Setting

Surprisingly, only 11 papers reported studies exclusively on active duty personnel. Three studies reported data from both military and non-military personnel [i.e., civilian law enforcement (27, 30, 36)], while six reported data on purely non-military personnel, including civilian personnel working in law enforcement and corporations or volunteers (26, 28, 38, 40, 41, 44).

Of the 20 published peer-reviewed studies on humans, 16 were conducted in training environments, three were conducted in corporate settings, and one was an online survey. Studies on military personnel have focused predominantly on service members attending Marine Corps or Army breacher training courses (29, 31–34, 37, 39, 43). Samples of law enforcement personnel included American Special Weapons and Tactics (SWAT) teams (28, 44) and Canadian Police (26). Four studies were conducted in non-training environments. These included an online survey of military and civilian law enforcement personnel (30), examination of the association between exposure to blast-induced impulse noise and irritability and anxiety symptoms at an explosives production company (38), and two examinations of noise-induced hearing loss among workers at an electrical transmission company, which the authors argued exposed workers to LLB similar in intensity and mechanism to that produced by firearms (40, 41).

In general, human studies on LLB exposure used relatively small samples in final analysis that ranged in size from 14 to 357, with an average of 83 participants. Of the five studies that included more than 100 participants, three reported data provided by civilian corporate employees (38, 40, 41), one was the web-based survey conducted by Carr et al. (30), and only one involved breachers (32). When only studies of military and civilian law enforcement training programs were examined, the average sample size was substantially smaller (~39 participants).

##### Exposures

In general, there was a notable amount of variability in the LLB exposures to which humans were subjected, as well as the detail in which it was described by authors, including a lack of information in some studies (35, 43). Several studies included examinations of LLB exposure from various weapons, including Howitzers (27, 36), the Carl Gustaf bazooka (28, 42), rocket-propelled grenades (42), light anti-tank weapons (42), shotgun door-breaching rounds (36), mortars (36), M4 Carbine rifles (36), and C4 (28). Training programs in which these studies were conducted ranged from 1 day (44) to 3 weeks (42), although most training courses were 2 weeks in duration (11, 26, 31–33, 35, 39, 43).

Several studies included measures of frequency of exposure to LLB, although the referent time frame varied across studies. Mean daily frequency of exposures was 10 in one study and 39 in another (38–44). Mean weekly exposures ranged between 40 and 50 per week (31, 38). In some cases, the frequency of exposures for instructors was reported as a calculated yearly metric and suggests that instructors experience hundreds of LLBs per year (29, 37, 39).

Estimated or measured overpressure exposure also varied. Several studies reported that exposures were generally below the 4 psi threshold thought to be safe from risk of tympanic membrane rupture (31, 37, 67). For example, some studies reported mean peak pressures ranging between 1 and 3 psi (28, 44), although one study reported mean peak pressures ranging between 4.8 and 8.5 psi (42). However, several studies reported that at least one subject who was exposed to overpressure that exceeded the 4 psi threshold, including exposure > 5 psi (33), 6 psi (37), 8 psi (32), 12 psi (36), and 13 psi (31). Only one paper reported total impulse pressure, which was measured at 7.5–19.4 psi per ms (42).

##### Outcomes

**Figure 2:**
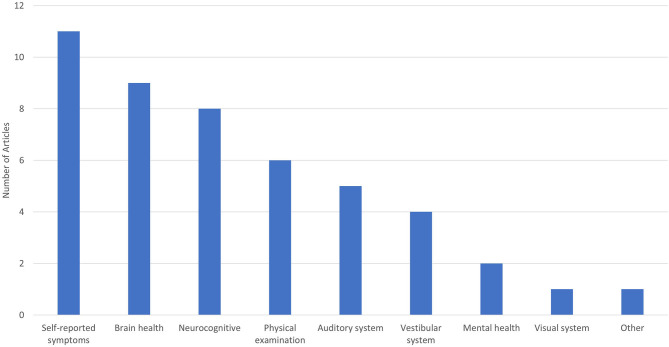
Outcomes assessed in human studies of low-level blast.

Studies of the effects of LLB on humans assessed a wide variety of outcomes (see Figure 2). Measures of self-reported symptoms (e.g., the Neurobehavioral Symptom Inventory, a collection of concussion-related symptoms) were most frequently collected, followed by objective markers of brain health (e.g., GFAP, ubiquitin carboxy-terminal hydrolase L1 [UCH-L1]), and assessments of neurocognitive function (e.g., the Automated Neuropsychological Assessment Metrics [ANAM], the Defense Automated Neurobehavioral Assessment). Five or fewer papers included assessments of the auditory, vestibular, and visual systems, mental health, or other outcomes (i.e., actigraphy to measure sleep health). Only six specific measures were used in three or more peer-reviewed papers: blast gauges (31, 33, 36, 42, 44), concussion-related symptoms (11, 30, 32, 33, 35), the ANAM (11, 31, 32, 35), UCH-L1 (11, 32, 35), GFAP (11, 27, 35), and distortion product otoacoustic emissions (37, 43, 44).

##### Summary of Findings From Human Studies of LLB

In general, results of studies of LLB suggest that it has potential to cause harm to humans. Specifically, several studies suggested the potential for adverse outcomes for brain perturbation (11), including axonal disruption (28), changes in neuromotor functioning (42), and impairments in working memory (31, 44). Furthermore, a few studies provided suggestive evidence for changes in biomarkers such as UCH-L1 (32), peripheral inflammatory markers (34), or APP (33). However, it is also worth noting that these effects were not necessarily replicated across studies and that Blennow et al. failed to demonstrate any neurochemical evidence of brain damage (27).

Beyond objective assessments of damage to the brain, several studies have also clearly indicated subclinical symptoms experienced because of exposure to LLB (11, 30, 33) and impulse noise generated by explosive blasts (38). However, four studies failed to show significant increases in self-reported symptoms as a function of exposure to LLB (26, 35, 37, 39). Two study teams attributed these failures to differences between instructors and students at baseline (26) and exposures that were generally below the 4 psi threshold and may have thus been considered safe (35).

Other studies have investigated the threat of LLB exposure on visual, auditory, and vestibular health. Taken together, studies to date have demonstrated potential adverse outcomes on the visual system, but not on the auditory or vestibular systems. Although there was only a single study directly examining adverse outcomes in the visual system, this study suggested that LLB can result in acute injury to the eye (29), which has the potential to affect warfighter performance, particularly for special forces operators (72). Hearing loss is often discussed as being a direct result of repeated exposure to loud noises such as those associated with overpressure exposure, but findings on the relationship between LLB and hearing loss were mixed. Studies by St. Onge et al. (43) and McBride and Williams (40, 41) suggest that exposure to LLB may result in hearing loss, but a study by Kubli et al. (37) did not. Regarding vestibular system functioning, although Carr et al. (32) showed deficits on two measures of vestibular system functioning, the other two measures they included did not show similar patterns. Research by St. Onge et al. (43) and Baker et al. (27) also did not show decrements in vestibular system functioning following LLB exposure during military and law enforcement breacher training courses, though the latter did show differences between instructors and students at baseline. In the one study that focused directly on vestibular system functioning of breaching instructors over 17 months and included a large battery of measures, Littlefield et al. (39) concluded that there were no adverse outcomes for vestibular system functioning as a result of LLB exposure. They did, however, demonstrate that there were differences in upbeat positional nystagmus for those with a history of mild TBI, the majority of which were presumably induced by HLB, and suggested that this could be an outcome of acute HLB, but perhaps not LLB (39).

### Limitations of Studies on LLB

#### Animal Studies

Although it appears to be the norm that papers presenting findings of the effects of LLB on animals do not discuss limitations of their work (46–50, 52–57, 59, 60, 62–65), a few articles addressed their limitations. These self-articulated limitations included concerns that the shock tubes used to generate LLB may not approximate the LLB exposure experienced by warfighters (45, 66), that adverse outcomes identified may not be reducible to purely primary blast (45), that pathophysiological processes that are apparent in animals may not necessarily translate to humans (66), and that these studies used small sample sizes (51, 66).

Beyond these self-identified limitations of animal research on LLB, several other limitations warrant further exposition. The first and perhaps most important limitation is that most of the animal studies investigating the effects of LLB exposure actually induced overpressure that exceeds the oft-discussed, yet arbitrary, 4 psi threshold expected to differentiate LLB from more intense blast exposure (28, 30, 32, 35–37, 42, 44, 67), and thus are not necessarily studies of LLB. Indeed, only 3 of the 23 articles on animals used overpressure exposure at or below the 4 psi threshold. This highlights the need for a clear discussion of thresholds for what constitutes LLB vs. what is no longer *low-*level blast.

Second, although animal models present an excellent opportunity to conduct basic science with the goal of understanding the complex processes resulting from LLB exposure, the animals or methods most frequently used may not be the best approximation for how such injuries or pathological processes may translate to humans (13). For example, there are remarkable anatomical differences between humans and most species used in animal research (80). This limits the ability to directly leverage the knowledge gained through these studies to inform clinical practice guidelines for the treatment of blast-induced injuries in humans at this time. However, this criticism is common in animal research that is aimed at understanding the processes involved in complex injuries and should not be taken as a reason to discount the animal literature entirely.

Third, although research sought to understand the effects of repeated exposure to LLB, the repetition of exposures was still quite limited, with the number of repetitions ranging from 2 to 12 exposures. Although these studies are valuable for understanding the effects of LLB exposure, this is not an appropriate approximation of repeated exposures that warfighters endure. For example, it is estimated that service members assigned to a 2–3 year tour as a breaching instructor will experience hundreds of exposures over the course of that specific tour alone (29, 37, 39). As a result, animal research on repeated blast exposure may not adequately inform our understanding of what our warfighters are truly facing, at least in some occupations.

#### Human Studies

Unlike animal studies of LLB, studies on humans regularly articulated a variety of limitations. The most articulated limitation was a limited sample size (11, 26, 28, 29, 31, 34, 37, 39, 40, 44), which typically occurred as a result of assessing LLB exposure in training environments. Studies on humans were generally quite small with an average sample size of ~83 for all included studies, and >40 for studies with data collected during military and law enforcement training programs. While data from instructors of such programs is particularly informative because they are suspected of having the largest cumulative exposure throughout their careers, the sample size of this unique subpopulation is meager and is frequently in the single digits in published studies.

Beyond limited sample sizes, there are several unique challenges associated with conducting research in warfighter training environments. First, these studies are primarily observational in nature. Second, such courses have intentionally low exposure over which the researchers have no control; such limited exposure may or may not result in adverse outcomes that are observable or quantifiable using existing measures, even if it has the potential to affect warfighter health and well-being over the course of their military careers (28, 37). Third, there can be remarkable variability in the LLB exposure of individuals within the same environment, and this can be challenging to assess at a person level rather than the overall event level (11). Fourth, the trainees and instructors in such courses that involve routine blast exposure are not randomly selected, and selection bias could limit the generalizability of these findings (30, 33). Furthermore, the study of training courses does not necessarily represent exposures incurred during less structured or regulated field training activities.

Several papers also articulated limitations associated with the study design or execution. For example, four papers noted the lack of a control group and/or appropriate controls (26, 32, 41, 42). Beyond this, several studies also highlighted that there is potential for an individual's personal history of blast exposure, head trauma, and/or extra-occupational activities (e.g., participation in sports) to influence the outcomes examined (26, 30, 35, 44), though such prior history was often not assessed. Additionally, some studies either did not include long-term follow-up or commented that their follow-up periods may not have been sufficient to observe differences in the outcomes of interest (28, 33, 42, 44). Two papers also articulated limitations associated with statistical procedures used (33, 40).

A number of studies described limitations associated with the measures they used. Some of these limitations related to the measures of blast exposure, such as an inability to articulate exposure at the individual person level (37), issues associated with the placement of blast sensors (32), and an inability to compare exposure levels with an analogous population (40). Another commonly mentioned limitation was that outcome measures often relied on self-report, which can be associated with recall bias and narrow ranges of reporting (e.g., for symptom reporting). Some researchers have also called for inclusion of more objective measures (11, 30, 40, 42, 67). A few other limitations, including those associated with missing measures [e.g., measuring certain additional biomarkers (32)] and difficulty in differentiating between commonly comorbid conditions (e.g., postconcussive syndrome and PTSD) when examining symptoms (30), were also discussed.

One additional limitation that was not discussed in the published studies of LLB using humans was a lack of assessment of blast exposure between training courses and follow-up. Although some studies collected follow-up data after the completion of training courses, limited efforts were made to account for additional exposures that may have occurred after the training but during the study window. Despite the plethora of limitations that afflict studies of LLB, our knowledge of the potential adverse outcomes of LLB has grown remarkably over time. Future research is and will continue to be needed to ensure an accurate understanding of the outcomes associated with overpressure exposure.

## Discussion

In this research, we sought to conduct a scoping review of peer-reviewed articles that reported investigations of the potentially detrimental effects of overpressure exposure, with a targeted focus on LLB exposure. Because terminology for LLB exposure is inconsistent at best, we used a comprehensive list of search terms regarding both HLB and LLB. After reviewing titles and abstracts from nearly 5,600 articles, we identified 3,215 articles that were relevant to blast (HLB and/or LLB) exposure. Most of these articles were focused on human subjects and often represented more than one subspecialty of medicine. This highlights the complexity of blast effects on human tissues and underscores the importance of interdisciplinary approaches to blast injury research.

Although research on LLB is growing rapidly, it still presents a tiny fraction of research on blast injury. Specifically, our review located only 51 peer-reviewed published articles on LLB across the past 20 years, including both animal and human research. Although animal studies have generally identified several adverse effects on complex physiological processes, conclusions based on human subjects' research are somewhat mixed regarding whether LLB exposure may be a threat to warfighter health and performance. Nonetheless, the results of this review support previous assertions that LLB exposure has the potential to harm warfighters (67, 70). The most well-supported adverse outcomes identified in humans remain self-reported symptomology (e.g., headaches, trouble hearing), particularly among those with greater cumulative exposure (e.g., breacher training instructors) (45, 70). However, given the diversity of the outcomes examined to date, much more research is needed to expand our understanding of the potential effects of LLB exposure, including examining whether adverse outcomes associated with repetitive LLB exposure present a threat to performance of duties and/or increased risk of adverse long-term health outcomes, including Alzheimer's disease (69, 72, 73).

Although this scoping review was conducted according to published guidelines to ensure methodological rigor (23), several limitations of this work should be noted. First, our review intentionally only included peer-reviewed articles published in English and may thus have missed some important ongoing research. For example, research conducted by those affiliated with the Department of Defense (DoD) is often published in the form of white papers, briefs, and/or technical reports outside of peer-reviewed outlets and thus would not have met our inclusion criteria. However, our focus on peer-reviewed publications was intentional and based on the premise that such research has been completed and was found to be of sufficient merit to warrant publication and dissemination among the broader scientific community. The intention of this review was to provide a summary of the literature that had been made publicly available to researchers and medical providers, both within and outside of the DoD, rather than including additional reports that may be limited to only DoD personnel. For further information on such studies (including work currently in progress), interested readers are referred to the summary provided by Simmons et al. (81).

Second, although we used a comprehensive list of search terms, it is still possible that some articles were not captured in our review. To offset this limitation, we intentionally used search terms that were not limited to purely LLB. Unlike other recent reviews of LLB (81, 82), our review evaluated the title and abstract of nearly 5,600 articles investigating blast to identify those that were relevant to LLB. Because of the lack of consistency in the terminology used to describe LLB within the scientific community, this was the only way to ensure that all relevant articles were captured in our review. We also took several additional steps to ensure that the search was as thorough as possible. Even so, we note that it is still possible that our review missed some peer-reviewed articles on LLB (e.g., because they used shock waves that we did not deem to be low-level) (83). Additionally, it should also be noted that because the field of blast research is rapidly evolving, even the most comprehensive literature search will rapidly become outdated as more articles are published. For example, in the year since we conducted our search, several additional papers on LLB been published summarizing epidemiological investigations (84, 85), case studies (86), studies of training courses (87), animal studies (88), and more.

It is important to note that two other literature reviews on LLB were published within the past year. Specifically, one review was prepared by researchers at RAND (81) as part of their efforts to facilitate the Seventh DoD State-of-the-Science Meeting on blast injury research, a conference held in 2018 that focused specifically on the neurological effects of repeated LLB exposure. This report specifically examined all research, including peer-reviewed and gray literature, that received funding from the DoD, was conducted at a DoD laboratory, and/or had a DoD-affiliated author. Our review included fewer articles than the RAND review, due in large part to their inclusion of gray literature and ongoing as well as completed work. While their review highlights more ongoing and newly emerging work, much of it is not accessible to the public and/or has not been peer-reviewed for scientific rigor. Additionally, LLB exposure is more common in military populations, but important research on LLB is being conducted in non-military populations by researchers who are not affiliated with the DoD, including researchers outside of the United States (71), as evidenced by the 17 articles included in our review that were not DoD affiliated in any way.

The second review was prepared by researchers at the Defense and Veterans Brain Injury Center and included only peer-reviewed published literature on the effect of LLB on humans (82). Given the important advances in animal research that is directly relevant to LLB in human populations, we believe the inclusion of these articles in a comprehensive review is essential because animal research allows for more experimental control and more comprehensive investigation than can ethically be conducted with humans. As research on LLB continues to advance, SMEs from these scientific disciplines will need to collaborate to translate findings from animals to human populations and will need to be informed about all relevant literature. Additionally, while Belanger et al. review included two articles that ours did not [both of which were technical reports rather than peer-reviewed publications (89, 90)], our review identified four additional articles (36, 38, 40, 41) investigating the effects of LLB on humans than theirs, likely due to rigorous methods to identify additional studies that were not revealed through our initial literature search.

## Conclusion

Due to the complexity of blast overpressure and its potential effects on the human body, a wide range of multidisciplinary expertise is required to identify prevention, mitigation, and treatment strategies for those exposed to blast overpressure. However, ensuring that emerging research is disseminated effectively to scholars from multiple disciplines has historically been a challenge. With this scoping review, we hope we have provided a clear, accessible overview of research on blast exposure that has been published within the past two decades, with a specific focus on LLB. It is intended to provide a detailed overview for researchers and medical providers from a variety of disciplines to increase awareness and understanding of the diverse studies on the topic. Research on the sequelae associated with blast overpressure exposure should and will continue to grow and develop over time, and it presents an important avenue for ensuring the health and well-being of members of the U.S. Armed Forces and others who are routinely exposed to blast.

## Data Availability Statement

The raw data supporting the conclusions of this article will be made available by the authors, without undue reservation.

## Author Contributions

JB led the research effort. SF and JJ coded data Levels 1 & 2. JB and SF coded data at Level 3. Manuscript was drafted primarily by JB and RE. All authors contributed to the theoretical framework of the paper, interpretation of results, contributed to the development of this manuscript, provided comments, and revisions.

## Conflict of Interest

The authors declare that the research was conducted in the absence of any commercial or financial relationships that could be construed as a potential conflict of interest.
