# Supplementary material for: Potential Health and Performance Effects of High-Level and Low-Level Blast: A Scoping Review of Two Decades of Research
Source: Front Neurol. 2021 Mar 10;12:628782. doi: 10.3389/fneur.2021.628782 (PMC7987950; doi:10.3389/fneur.2021.628782)
Supplement: Supplementary file 1 [file Table_1.DOCX]

**Supplementary Material**

**Data form used for level 3 data extraction.**

1. What is the full citation of the article?
2. Did the article discuss chronic, low-level overpressure? Yes/No
   1. Skip logic: If no, the record will close out.
   2. Display logic: If yes, what was the purpose of the article? (Open text) Please use direct quotes (1-2 sent.) from abstract and include page number(s).
   3. Was chronic, low-level overpressure explicitly defined? Yes/No
   4. Did the article also discuss acute, high-level overpressure? Yes/No
   5. Display logic: If chronic, low-level overpressure was discussed, what terminology was used in the abstract? (Open text, List each term.) Also, please indicate whether each term was defined in the text. If it was defined, please provide a direct quote of the definition and the page number(s). NOTE: Terminology in the abstract should not also be counted in the rest of the article.
   6. Display logic: If chronic, low-level overpressure was discussed, what terminology was used throughout the article? (Open text, List each term.) Please indicate whether each term was defined in the text. If it was defined, please provide a direct quote of the definition and the page number(s).
   7. Display logic: If acute, high-level overpressure was also discussed, what terminology was used in the abstract? (Open text, List each term) Also, please indicate whether each term was defined in the text. If it was defined, please provide a direct quote of the definition and page number(s). NOTE: Terminology in the abstract should not also be counted in the rest of the article.
   8. Display logic: If acute, high-level overpressure was also discussed, what terminology was used throughout the article? (Open text, List each term) Also, please indicate whether each term was defined in the text. If it was defined, please provide a direct quote of the definition and page number(s).
3. What type of article was it? Empirical/Nonempirical
   1. Skip logic: If nonempirical, skip to nonempirical questions.
   2. Display logic: If empirical, what was the hypothesis of the study? (Open text) Please use direct quotes (1-2 sent.) and include page number(s).
   3. What type of subjects were included in the study? (Human, Animal, Other)
      1. Display logic: If animal, what kind? (Open text)
   4. How many subjects were included in the study? (Open text)
   5. Were the study subjects exclusively active duty military personnel? (Yes/No)
   6. What was the study setting? (Operational, Training, Laboratory, Other)
   7. Did the researchers have control over the exposures? Yes/No
   8. Were the subjects randomly assigned to a condition? Yes/No
   9. Please describe the subjects in as much detail as possible. (Open text – Breakdown of subjects [e.g., “11 soldiers, 4 engineers”], Age, Rank, etc.)
   10. Please describe the study exposures in as much detail as possible. (Open text – e.g., Frequency of blasts, Intensity of blasts [e.g., psi], Distance from blasts, Amount of blasts [e.g., pounds of explosives used], Environmental features of blasts [e.g., Land, Water, Open space, Closed space], any personal protective equipment [PPE] used, etc.)
   11. Using instrument names, please describe the study outcomes in as much detail as possible? (Open text – Acute, Cumulative, Epidemiological, Medical, Neurocognitive, Specific biomarkers [e.g., tau], Self-reported symptomology [e.g., headaches], etc.)
   12. What were the study results? (Open text) Please use direct quotes (1-2 sent.]) and include page number(s).
   13. Did the researchers collect data relevant to overpressure mitigation strategies? (Yes/No)
   14. Did the researchers collect data relevant to personal protective equipment (PPE)? (Yes/No)
   15. What was the study conclusion? (Open text) Please use direct quotes (1-2 sent.) from abstract and include page number(s).
   16. What were the study limitations? (Open text – bulleted list) Please use direct quotes (1-2 sent.) and include page number(s).
   17. Display logic: If nonempirical, what type of article was this? (Open text – Systematic literature review, Meta-analysis, Clinical review, Letter to Editor, etc.)
   18. Display logic: If systematic literature review, how did the authors review the literature? (Open text – e.g., Databases, Search terms, etc.)
   19. What was the study conclusion? (Open text) Please use direct quotes [1-2 sent.] and include page number(s).
4. Please include any other important details in the box below.
